# Complete genome sequence of *Bacillus velezensis* strain S-LA5, an endophyte isolated from the medicinal plant *Leucas aspera*

**DOI:** 10.1128/mra.00550-26

**Published:** 2026-09-30

**Authors:** Md. Shahrear Parvaj Sujon, Sharmin Islam, Abdullah Al Kafi, Avijit Pal Apu, Dipali Rani Gupta, Tofazzal Islam

**Affiliations:** 1 Institute of Biotechnology and Genetic Engineering (IBGE), Gazipur Agricultural University (GAU) 198780, Gazipur, Bangladesh; Montana State University, Bozeman, Montana, USA

**Keywords:** complete genome, Oxford Nanopore sequencing, probiotic, *Bacillus*, endophytic bacteria

## Abstract

This study reports the complete genome sequence of *Bacillus velezensis* strain S-LA5, isolated from *Leucas aspera* in Bangladesh. Oxford Nanopore sequencing yielded a 4.1-Mbp genome (46.5% GC) in a single contig, predicted to encode traits for nutrient acquisition, plant growth promotion, and biocontrol, underscoring its potential in sustainable agricultural disease management.

## ANNOUNCEMENT

*Bacillus velezensis* is a Gram-positive bacterium found in soil, rhizosphere, and agricultural habitats. It associates with plants as epiphytes or endophytes and is renowned for growth-promoting traits, including phosphate solubilization, siderophore release, and nitrogen fixation (1, 2). Endophytic *B. velezensis* strains, including Lle-9, Amfr20, and JRX-YG39, exhibit growth-promoting and broad antifungal activity, suppressing pathogens like *Fusarium oxysporum*, *Botrytis cinerea*, *Rhizoctonia solani*, and *Alternaria alternata* (3–5).

We report the complete genome of *Bacillus velezensis* strain S-LA5, isolated from surface-sterilized floral tissues of *Leucas aspera* collected 23 May 2025, in Birol Upazila, Dinajpur, Bangladesh (25.63° N, 88.55° E). One gram of flowers was collected from multiple plants and sterilized with 70% ethanol for 1 min, followed by 2% sodium hypochlorite for 3 min, rinsed thrice in sterile distilled water, and then aseptically macerated. The homogenate was serially diluted to 10⁻^5^ and 100 µL plated on nutrient agar (Liofilchem, Italy). Distinct colonies were purified by three successive subculture steps and incubated aerobically at 28°C for 48 h. A single colony was sub-cultured in nutrient broth (Liofilchem, Italy) and incubated at 28°C for 48 h, 180 rpm. Genomic DNA was extracted using the Tissue Genomic DNA Extraction HE Mini Kit (FAVORGEN, Taiwan) and quantified with a Qubit 4.0 fluorometer (Thermo Fisher Scientific). Sequencing libraries prepared with Oxford Nanopore Technologies kits (SQK-NBD114.96, Barcoding Kit 96 V14) were sequenced on a MinION R10.4.1 flow cell, following the manufacturer’s protocol. No DNA shearing or size selection was performed. Base-calling was performed using Dorado v1.2.0 (SUP model).

Sequencing generated 30,107 reads totaling 262,233,383 bp raw reads (N_50_ 15,327). Reads were quality-checked with NanoPlot v1.46.1 (6), adapters trimmed via Porechop v0.2.4 (7), and reads filtered (>1 kb) using NanoFilt v2.8.0 (8). *De novo* assembly was performed with Flye v2.9.5 (9) and polished with Racon v1.5.0 (10) and Medaka v2.0.1 (11). Genome quality was assessed with QUAST v5.2.0 (12), and completeness was assessed with CheckM v1.2.4 (13) using the *Bacillus* CheckM marker set. Annotation was performed with the National Center for Biotechnology Information (NCBI) Prokaryotic Genome Annotation Pipeline v6.10 (14) and the RAST server v2.0 (15). For all software, default parameters were used except where noted.

The *B. velezensis* strain S-LA5 comprises a 4.1 Mbp genome with 1 contig, 46.5% GC content, and 319 subsystems (metabolic features). NCBI annotation revealed 3,884 coding sequences (CDSs), including 3,812 protein-coding genes, 86 transfer RNAs (tRNAs), and 27 ribosomal RNAs (rRNAs) (Table 1). NCBI average nucleotide identity (ANI) analysis confirmed the closest similarity to *B. velezensis* (ANI 99.15%, assembly coverage 94.26%, type assembly coverage 94.83%; GCA_001267695.1) (16).

**TABLE 1:** Genomic features of *B. velezensis* strain S-LA5 isolated from the tissues of *L. aspera* flowers

Genome feature	S-LA5
SRA	SRR37520456
GenBank accession	JBTKTL000000000.1
BioSample accession	SAMN54376226
16S accession	PX884500
GenBank assembly	GCF_055690625.1
Assembled genome size (bp)	4,062,983
GC content (%)	46.5
Coverage (×)	59.0×
Genome completeness (%)	99.41% (CheckM)
Contamination (%)	0
N_50_ value (bp)	4,062,983
Number of contigs	1
Genes (total)	4,002
Coding sequences (CDSs)	3,884
CDSs (with protein)	3,812
CDSs (without protein)	72
Genes (RNA)	118
rRNAs	27 (9 × 5S, 9 × 16S, 9 × 23S)
Complete rRNAs	27 (9 × 5S, 9 × 16S, 9 × 23S)
tRNAs	86
ncRNAs	5
Pseudogenes (total)	71
Pseudogenes (ambiguous residues)	0
Pseudogenes (frameshifted)	43
Pseudogenes (incomplete)	40
Pseudogenes (internal stop)	7
Pseudogenes (multiple problems)	15
Number of subsystems (metabolic features)	319

Analysis using BAGEL4 (17) detected antibacterial bacteriocin and RiPP clusters encoding amylocyclicin, LCI, and plantazolicin. AntiSMASH v8.0.4 (18) predicted 14 secondary metabolite gene clusters, including surfactin, bacillaene, fengycin, plantazolicin, bacillibactin, and bacilysin. Predictive complete nonribosomal peptide synthetase (NRPS) operons for surfactin (srfAA-AD) and pliplastin (ppsA-E), highlighting lipopeptide-mediated antifungal potential (19). Genes predictive of cell wall-degrading enzymes (*csn*, *bglS*, *eglS*, and *bpr*), exopolysaccharide biosynthesis (*epsD* cluster), and phytase (*phy*) suggest root colonization and phosphorus mobilization (20). Collectively, these genomic traits predict strain S-LA5 as a candidate for biocontrol and plant growth promotion in sustainable agriculture, though functional contributions require experimental validation.

## Data Availability

The whole-genome sequence of *Bacillus velezensis* strain S-LA5 has been deposited in NCBI GenBank (JBTKTL000000000.1), GenBank assembly accession (GCF_055690625.1), and the Sequence Read Archive (SRA) (SRR37520456) under BioProject (Mining Bangladesh Biogold) accession PRJNA1055760. Additional annotations generated using RAST, BAGEL, and antiSMASH have been deposited in Zenodo and are accessible at https://zenodo.org/records/19602378.
